# Formal modeling and analysis of security schemes of RPL protocol using colored Petri nets

**DOI:** 10.1371/journal.pone.0285700

**Published:** 2023-08-17

**Authors:** Farooq Ahmad, Muhammad Tayyab Chaudhry, Muhammad Hasan Jamal, Muhammad Amar Sohail, Daniel Gavilanes, Manuel Masias Vergara, Imran Ashraf

**Affiliations:** 1 Department of Computer Science, COMSATS University Islamabad, Lahore Campus, Lahore, Pakistan; 2 Universidad Europea del Atlántico, Santander, Spain; 3 Universidad Internacional Iberoamericana, Campeche, México; 4 Universidad Internacional Iberoamericana, Arecibo, Puerto Rico, United States of America; 5 Universidade Internacional do Cuanza, Cuito, Bié, Angola; 6 Fundación Universitaria Internacional de Colombia, Bogotá, Colombia; 7 Department of Information and Communication Engineering, Yeungnam University, Gyeongsan, Korea; University of Jeddah, SAUDI ARABIA

## Abstract

In the Internet of things (IoT), data packets are accumulated and disseminated across IoT devices without human intervention, therefore the privacy and security of sensitive data during transmission are crucial. For this purpose, multiple routing techniques exist to ensure security and privacy in IoT Systems. One such technique is the routing protocol for low power and lossy networks (RPL) which is an IPv6 protocol commonly used for routing in IoT systems. Formal modeling of an IoT system can validate the reliability, accuracy, and consistency of the system. This paper presents the formal modeling of RPL protocol and the analysis of its security schemes using colored Petri nets that applies formal validation and verification for both the secure and non-secure modes of RPL protocol. The proposed approach can also be useful for formal modeling-based verification of the security of the other communication protocols.

## Introduction

The Internet of things (IoT) has provided the notion of a smartly connected world. IoT is a system in which gadgets, computing machines, or mechanical devices with the capabilities of sensing, identifying, controlling, and responding, are interconnected to enable service provisioning in which humans have little involvement. The main objective of fostering IoT is to develop a better world for humans [1, 2]. Thus, for better interaction with cyber-physical devices, it is important to empower them with their own source of collecting data or information, so that these intelligent devices can monitor the happenings of real-world [3]. While wireless sensor networks (WSN) and cloud computing are important enabling technologies for IoT, the world is witnessing fast development. In the recent past, we have seen the proliferation of IoT in breadth and depth such as smart homes, smart factories, smart healthcare, surveillance, and miscellaneous applications [4–6]. Low power and lossy networks (LLN) are those in which the connected devices are characterized by limitations in the context of resources such as memory, processing power, and battery, along with a high rate of packet loss and low data rate. LLN is widely used in WSN and it is inherently divergent from typical networks as these networks have several restrictions. An additional facet of the said networks is the high volume of data transmission, which often results in network congestion [7, 8]. Such situations make these systems undergo data loss and transmission postponements. Thus, to maintain the real-time application of the LLN, the above-mentioned shortcomings must be managed during the development of LLN routing protocols [9]. Routing protocols for LLN (RPL), use destination-oriented directed acyclic graphs (DODAG) for building network routes consisting of nodes (devices) and use IPv6 messages (ICMPv6) [10]. Five different main types of RPL control messages are defined for RPL. These are used for network discovery, propagating information, security, and checking inconsistencies in the network. The traffic flow of the network can go in two directions: upwards to the root node of the acyclic graph or broadcasted downwards.

Formal modeling of a system is a design technique that uses standard mathematical models to develop hardware and software systems for detailed analysis before building the actual system. Formal methods leverage mathematical proof to complement system testing which is carried out to ensure correct behavior. With systems getting complicated, safety becomes more crucial and important. In this regard, the formal modeling approach provides additional insurance for the system’s desired behavior [11]. IoT applications are vulnerable to various security threats [12]. These threats may be related to availability & privacy, physical, multi-stage advanced persistent threats, and malware [13, 14]. Modeling a system can give a deep insight into the aspects which may have been ignored otherwise. However, one of the limitations of using formal techniques to model large-scale complex systems is the readability and understandability of the formal model of the system. The formal model of a complex system can become so large and complex that may lower its understandability. Furthermore, formal analysis and validation of large-scale formal models of the systems are challenging and face the famous state-space explosion problem [15].

Petri nets are a prominent formalism for modeling and analyzing concurrent and distributed systems. There are many types and classes of Petri nets presented in the literature which include, place-transition nets, timed Petri nets, stochastic Petri nets, or Fuzzy Petri nets, which have limited modeling power. Colored Petri nets (CPN) are high-level nets that have the power to use different data types, time, probability, and priority, which reflect more modeling power. Furthermore, CPN ML language is used to model the complex systems which are based on the functional programming language i.e. Standard ML (SML). Furthermore, one of the main features of CPN is that it supports a hierarchy that helps develop modules for large-scale systems. Therefore, the main limitation of modeling large-scale systems can be tackled through the colored Petri nets.

Formal analysis of the wireless networks (through Petri nets) at the protocol level is a growing research area. CPN is a graphical language that is used for modeling concurrent systems for analysis [16]. Typical applications include the modeling and security analysis of communication protocols, data transmission analysis, distributed systems (including Cloud Computing, Edge Computing, and the IoTs), manufacturing systems, and business process modeling. In short, the CPN models can incorporate the phenomenon which can be described in terms of the states of the system and the events or transitions that can cause the change or transition in the state of the system. Hence, the CPN modeling can be used to simulate, analyze and debug a system and thus improve the working and behavior of the system in terms of time consumption, throughput, fault tolerance, and economy of cost [17].

Therefore, to accomplish the widely acknowledged lack of formalization in the domain of security and privacy issues in IoT, a colored Petri net-based RPL secure model for specification, analysis, and validation is presented in this paper. Furthermore, for our proposed work, to the best of our knowledge, we have performed the novel modeling of routing protocols of IoT environments. We have provided the base for the security analysis of RPL protocol and possible extension of RPL class of algorithms. The main challenges in modeling RPL (as per the scope of work) include the representation of DODAGs, control messages, the behavior of every wireless node according to control messages, and the secure and unsecured modes of the RPL, communication, and bootstrapping of the algorithm. These challenges have been addressed in this paper. This study is related to the following streams of research.

- Formal modeling and techniques for the evaluation of security models for IoT routing,
- Formal specification and validation of the security models related to routing in IoT and analysis of security level of IoT protocols,
- Identifying potential adversaries and improving the execution of current defensive protocols by Petri nets theory,
- Evaluation of possible enhancement in the efficiency of security technique in terms of robustness, the volume of messages exchanged, complexity, and privacy.

The rest of the paper is organized as follows. Related work for IoT-based systems is presented in Section 2. Section 3 presents the introduction of Petri nets while preliminaries for RPL and LLN are discussed in Section 4. Section 5 discusses the proposed CPN-based modeling of the RPL protocol. Section 6 presents the results and discussions regarding CPN-based modeling while section 7 concludes the paper.

## Related work

The lack of formal models and the heterogeneous nature of security and privacy issues in IoT research is the main cause of the difficulty to evaluate new security models in IoT [18]. IoT could be a giant network of interrelated devices that may dispense data about the surrounding. Z-wave, ZigBee, Bluetooth, long-term evolution (LTE) (data link protocol), RPL, CORPL (network layer), IPv6 over Bluetooth low energy, 6lowpan (network layer encapsulation protocols), etc. are different protocols of IoT at different layers. Initially, we are very much interested in two important and useful protocols of IoT at the network and its upper layer respectively. 6LowPan is a widely used protocol that works along with RPL and provides services of encapsulating IPv6 long header on small data up to one hundred and twenty-eight bytes. The protocol helps to compress the header to overcome transmission cost and additional functionality of fragmentation of meeting the low limit of data and provides multi-hop too. On the other hand, RPL at the network layer is a protocol based on the destination-oriented directed acyclic graph (DODAG) and IPv6, in which setup devices have constraints on energy, computational power, and memory. RPL supports p2p as the network expands when a new node wants to be the part of existing DODAG according to its distance vector [19]. Sybil attacks, forwarding attacks, sinkhole attacks, flooding attacks, wormhole attacks, rank attacks, etc. are some of the attacks on the RPL which can strike vulnerable networks. Butun et al. investigated security threats and attacks related to these two protocols [20]. To accomplish a system, many mitigation techniques are proposed to tackle these attacks by the researchers [21–23].

### Formalization to IoT based systems

Security issues in IoT systems and solutions for those challenges is an ever-growing domain for researchers, surveys on IoT-based systems can be found in studies [21–25]. Tata found a way to decompose a process-aware application system through Petri nets using the expansion of the Node-RED tool [26]. Model-driven engineering was first introduced by Romina Spalazzese and Federico Ciccozzi. Through MD4IoT they demonstrated the model of an intelligent and self-adaptive IoT system [27]. Boytsov et al., [28] proposed formally analyzed, and formally verified pervasive computing. In another research, the authors demonstrated how existing formal methods can be extended for ubiquitous and pervasive computing and addressed the issue of static and dynamic verification. Research like [29, 30] focused on designing time formal modeling and verification. Wenbo Wang and Yingfeng Zhang used hierarchal-based color Petri-nets with intelligent tokens that can behave like smart objects. They used the decision tree method for exception handling. In a case study of the manufacturing industry, simulation results claimed that the approach can be used for real-time systems [31]. Complex models can be found in these articles [32–35] of smart, complex, intelligent, and self-adaptive systems. These studies applied formalism to IoT-based embedded systems [36, 37] and a special field of health care [38].

### Modeling and analysis of IoT regarding security

IoT is an emerging technology and also the future of many critical fields like blockchain technology; [39] research proposed the possible solutions in the blockchain. In recent research on the composite field of simulation & modeling and IoT, authors gave semantics to IoT-based systems’ components. They proposed a model checker named PRISM to check if the system is working properly [40]. In this research, the authors performed the formal modeling of another IoT protocol MQTT and analyzed the system statically. They claimed that their model perfectly works for the first two quality of services but put an error against the third quality of service and, hence, they suggested a chance of enhancement on the specification level of the protocol [41]. The research in [42] proposes a Petri net-based modeling methodology and optimization algorithm for collaborative business processes in IoT applications and solves the problem of sensing event scheduling. This study does not consider the RPL-based security scheme in IoT.

Another line of related work is IoT checker, a new ontology-based security approach to tackle the configuration problem of IoT devices. Various configurations are weak at different risk levels. Mujahid and Muhammad introduced an IoT risk analyzer for the formal analysis of those risks quantitatively. It takes configurations, weakness score, and adversary abilities as input [43]. A novel technique to deal with examining the security of a digital framework (CPS) is suggested. The proposed methodology is connected to a completely working water treatment and a testbed was built. The authors analyzed attacks experimentally and suggested several research problems for IoT-based systems having the same infrastructure [44]. Formal methods from modeling to simulation are applied as an agent-based Petri net to check the correctness of the already proposed model [45]. On a very recent note, the authors formally defined and mathematically proved each attribute in the traffic decorrelation process. They lessened the end-to-end delay by 30% and communication overhead by 50%. In particular, they split the whole WSN into least associated dominating sets which work in the round-robin form. As a result, they were able to reduce the number of active traffic sources per unit of time, while giving access to any node in the WSN [46]. Using colored Petri nets, the authors captured the mobility of nodes with no decay in speeds to an extent. They formalized mobile nodes in two different layers through hierarchal models. Further [47], provides a study of RPL, its attacks, and mitigation methods, including a review of the RPL standard, a classification scheme for mitigation methods, and a discussion on RPL-based intrusion detection systems and does not consider the formal approaches.

## Introduction to Petri nets

Petri nets are weighted bipartite graphs that are capable of formal modeling any kind of system. Petri nets are used to ensure completeness and to improve the correctness of the design. Further, such kind of formalism helps to avoid ambiguity and incompleteness in the design at the designing phase of the system development life cycle [48]. Petri nets are usually used to model communication protocols, data networks, distributed, etc.

Petri nets are a prominent formalism for modeling and analyzing concurrent and distributed systems. There are many types and classes of Petri nets presented in the literature which include, place-transition nets, timed Petri nets, stochastic Petri nets, or Fuzzy Petri nets, which have limited modeling power. Colored Petri nets (CPN) are high-level nets that have the power to use different data types, time, probability, and priority, which reflect more modeling power. Furthermore, CPN ML language is used to model the complex systems which are based on the functional programming language i.e. Standard ML (SML). Furthermore, one of the main features of CPN is that it supports a hierarchy that helps develop modules for large-scale systems. Therefore, the main limitation of modeling large-scale systems can be tackled through the colored Petri nets.

There are two types of vertices in the Petri net graph which are called *places* and *transitions*. The places represent the state of the modeled system and transitions are used to represent the operations of the system. Arrows connecting both places and transitions are called arcs. Moreover, colored Petri net (CPN) based formalism combines the strength of Petri nets [11] with the Standard ML (SML) [48] which is suitable for primitives for the definition of data types and manipulation of data. Furthermore, CPN tools [49] provide a graphical environment to create, edit, simulate, and analyze CPN models; it is widely accepted by the research community and it can also visually divide the hierarchical components of the model. For comprehension, CPN supports different levels of abstraction which set the basis for hierarchal colored Petri nets. Hierarchal color Petri nets are used for the modeling and analysis of complex systems. For analysis of the modeled system, state space can be generated which explains the behavior of a specific system. In addition, simulation analysis of a particular model can give more insights into a system.

## Preliminaries of LLN and RPL

In this section, a brief introduction to the RPL protocol is included. The RPL protocol is an IPv6 routing protocol for low-power and lossy networks. LLN, such as sensors or radio networks typically do not have a predefined topology. A huge number of applications of lossy networks could be found in the sensor network. RPL protocol organizes the topology as a directed acyclic graph. It uses RPL IPv6 control messages to exchange data over the network [8], formal specification of these messages is given in Section 5. The word RPL itself is derived from routing protocol for LLNs and represents a distance vector based on destination-oriented acyclic graphs which are simply called DODAGs. Before going into how it works, one must be familiar with the key concepts which are at the heart of this routing protocol as given below. Directed acyclic graph, parent and child, root, DODAG, objective function, RPL instance, rank, DODAG ID, DODAG version number, storing and non-storing are different terms that are used in RPL theory; formal specification of these terms is given in Section 5.

### Our subset of RPL protocol

The RPL protocol is very large and detailed, it is feasible to focus on a small subset of the protocol as security concerns. The focus areas of this research are mainly on the two control message types i.e., a DODAG information object (DIO) and destination advertisement object (DAO) which play a vital role in the security of RPL protocol as these are the entry points of interaction with the other nodes.

### How destination oriented directed acyclic graphs are formed

**Fig 1:**
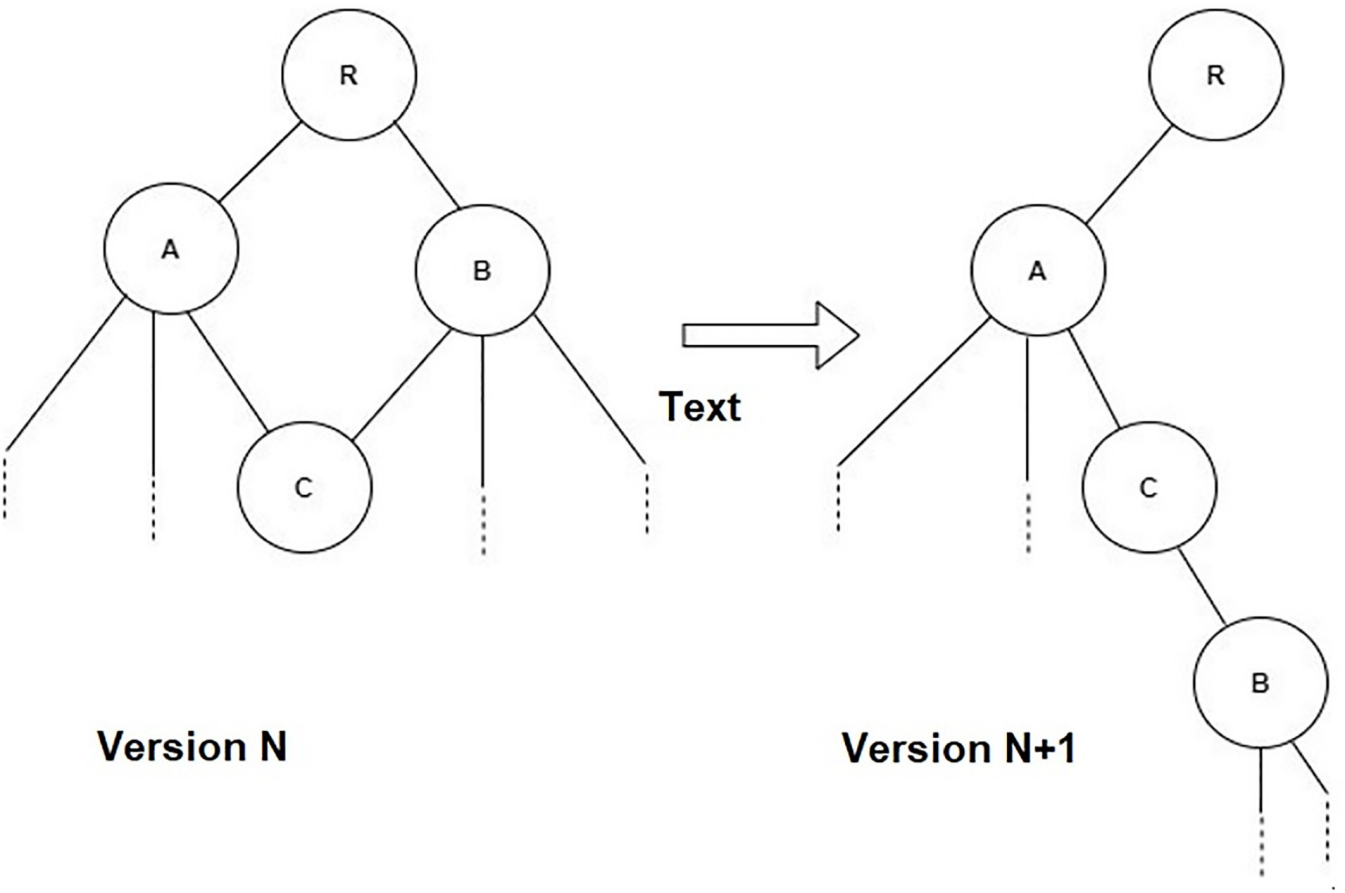
Increase in DODAG version number.

An instance of a routing protocol can have single or many DODAGs [50]. A DODAG typically only has one root node, and in the case that there are multiple root nodes defined, it is assumed that they all are pointing to the same parent node, i.e., a common backbone. The flow of the network can go in two directions, both down and up in the graph. Usually, the specific acyclic graph has one root node and the child can have one parent whereas the parent can have n number of children. A DODAG is uniquely recognized by the group of a DODAGID, which is the identifying number of the DODAGs (unique identifier root node), and RPL Instance ID (unique identifier of a network). The DODAG also contains a countable number of nodes (devices) that must have a rank associated with them, as well as a DODAG version number. DODAG version number is actually the current iteration number of that particular DODAG. The objective function allows a node to pick up the best-suited parent based on these factors like lowest rank, highest DODAG version number, and minimum distance [51]. Fig 1 shows how the DODAG changes over time.

The topology changes when nodes disconnect or when new nodes join the network. When an inconsistency is discovered, the DODAG version number is increased, and the nodes gradually update their routing tables. Because it is power and resource-consuming, to keep the routing table up to date continuously, the nodes only update their respective routing tables when a mismatch in the locally stored DODAG version number and the version number of neighbors is discovered. By detecting a mismatch using this method the inconsistency does not have to be broadcasted to all the nodes in the network but will be gradually updated as connected nodes discover that the information they have is of an older version. The DODAGID, RPL instance ID, DODAG version number, and the rank of a node make up the RPL identifiers. The RPL identifiers are used to identify, manage, and maintain a topology.

## CPN-based modeling of RPL

### Modeling of the RPL protocol of unsecure mode

The role of abstraction is critical in using any mathematical modeling language for the description of network systems [52]. The research proceeds towards the CPN modeling of RPL protocol of unsecure mode and then moves forward to the formal modeling and analysis of security schemes of RPL protocol using colored Petri nets. In this research, the focus is on transforming the CPN model into a network language, and for this purpose formal model of RPL protocol is used. To this end, the model is kept quite simple and does not dive deep into the lower level of abstraction. But to model the security aspects of this protocol, one must have access to the level of ‘control messages abstraction’. Moreover, the accurate translation of control messages into CPN language, containing places, transition inscriptions, and arc expressions, is an essential part of this study. Thereby, this study formally proves the existence of the unsecure and secure mode (also called the preinstalled mode) of the RPL protocol.

### Towards modeling of secure mode of RPL

**Fig 2:**
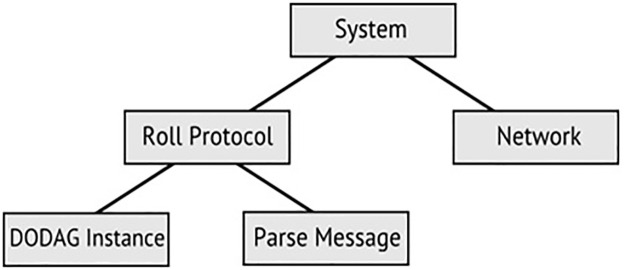
Hierarchy of the proposed model.

**Fig 3:**
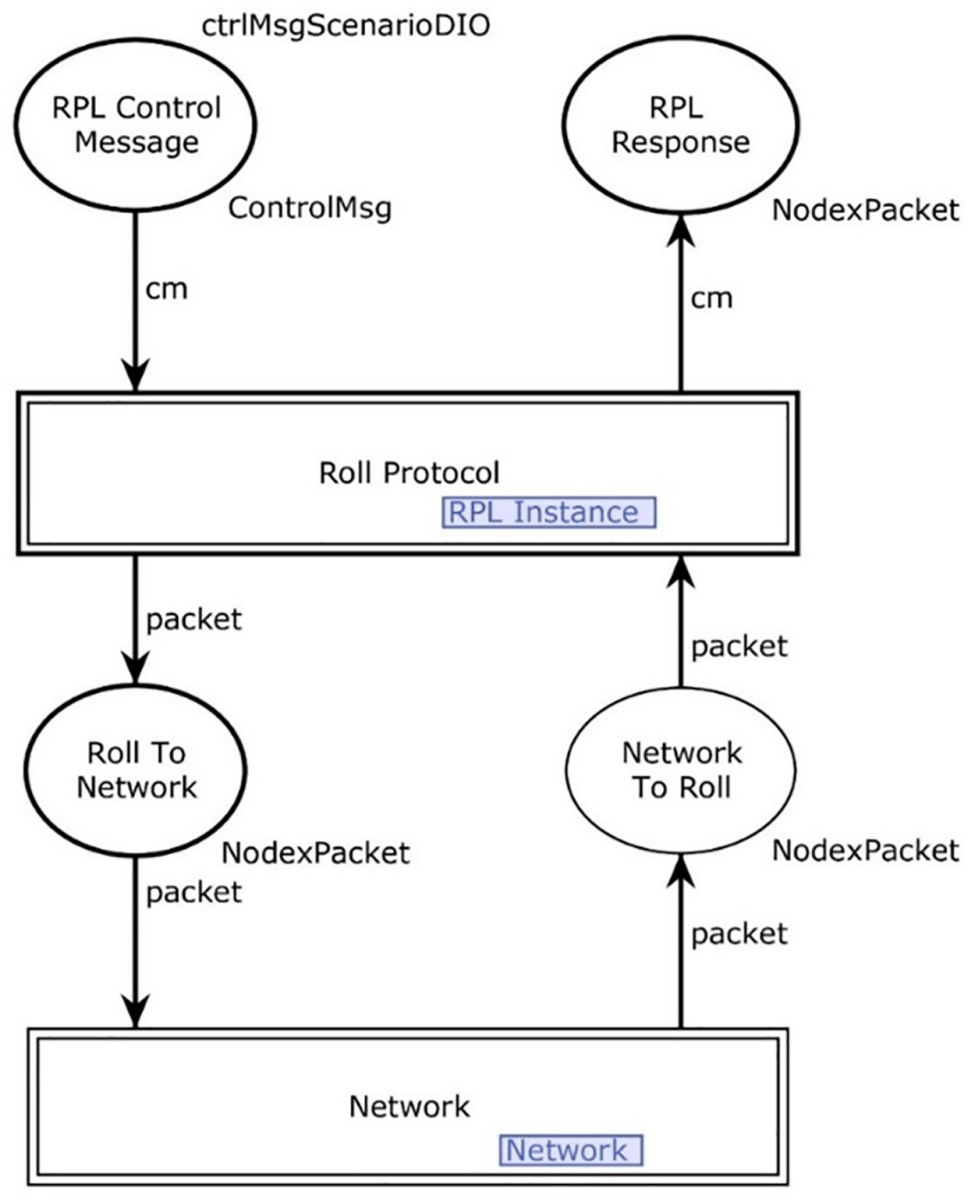
The main module.

Fig 2 shows the hierarchical view of the proposed system which contains five major modules. The main module has two transitions i.e., network and roll protocol (see Fig 3). Further, roll protocol transition deals with the actual behavior of the protocol. The IETF ROLL working group defined different standards according to application-specific routing requirements. In this research standards for RPL defined by RFC 6550 [8], are used as predefined rules for the RPL protocol for the modeling. Every set of rules is elaborated with its formal meaning. Therefore, the understanding of this study is associated with the mapping of RPL standards into the CPN description.

### Declaration of model

In this section, a brief description of color sets, variables, and use case scenarios are given.

#### Color Sets

All the color sets related to five control messages of the RPL protocol are defined in Table 1.

**Table 1:** Color sets used for the model.

Color set	Description
colset K = bool;	DIO Redundancy Constant
colset R = bool;	Rank Error
colset D = bool;	InstanceID predicate
colset Status = int;	Status of the Node
colset DODAGPref = int;	DODAG preference
colset DestinationCounter = int;	Counts destination for a specific node
colset DODAGID = int;	Unique identifier of DODAG
colset Flags = int;	Value of flag, destination or source
colset MOP = int;	Mode of operation
colset Rank = int;	Value of rank or relative position
colset VersionNumber = int;	Version number of the DODAG
colset Type = int;	CM type
colset Code = int;	Code part of a control message
colset RPLInstanceID = int;	Identifier of an instance
colset NO = int;	Number color set
colset Grounded = bool;	Check if goal has been met
colset DTSN = int;	One of the DODAG value
colset Algorithm = int;	Encryption algorithm
colset KIM = int;	Key values checks if signature values used in control message
colset Reserved = int;	8-bit unused field
colset Resvd = int;	3-bit unused field
colset LVL = int;	Security level
colset DODAGVersionID = NO;	Version number of the DODAG
colset Base = string;	Control message part
colset Options = string;	Options for DAO messages
colset Counter = string;	Message counter
colset Checksum = string;	A part of control consistency checks
colset KeyIdentifier = string;	2-bit field to check explicity
colset CCNonce = string;	Part of consistency check message
Closet DAOSequence = int;	Sequence of DAO messages

#### Formal specification of RPL messages

The parameters defined by the IETF role working group are used for the configuration of RPL control messages and are given in Table 2. All the control messages used in a model are made up of the DIO base object and base object header.

**Table 2:** Color sets related to RPL message.

Color set	Description
DODAG Information Solicitation	colset DISBaseObject = record flags:Flags * reserved:Reserved * options:Options;
DODAG Information Object	colset DIOBaseObjectHeader = record rplInstanceID:RPLInstanceID * versionNumber:VersionNumber * rank:Rank* g:Grounded * mop:MOP * dodagPref:DODAGPref * dtsn:DTSN * flags:Flags * reserved:Reserved;
	colset DIOBaseObject = record header:DIOBaseObjectHeader * dodagid:DODAGID * options:Options;
Destination Advertisement Object	colset DAOHeader = record rplInstanceID:RPLInstanceID * k:K * d:D * flags:Flags * reserved:Reserved * daoSequence:DAOSequence;
	colset DAOBaseObject = record header:DAOHeader * dodagid:DODAGID * options:Options;
DAO-ACK	colset DAOACKBaseObject = product RPLInstanceID * D * Reserved * DAOSequence * Status * DODAGID * Options;

DODAG Information Solicitation (DIS) is one of the control messages that flow in RPL infrastructure. Further, DIS-based objects contain three parameters namely, flags, reserved, and options. Therefore, DODAG Information Solicitation is a structured color set of record type containing three fields i.e. Flags, Reserved, Options, and this color set is constructed using the product of these three fields (for their detail, see Table 1). DODAG Information Object (DIO) is another control message which discovers the RPL instance. The parameters used for their configuration are as follows; RPL instance ID, Version number, Rank, Grounded (G), Mode of Operation (MOP), Preference (Prf), Flags, Reserved, and Options (see Table 1 for their explanation). First, the color set for the DIO object header is defined, and then the color set of the DIO-based object is defined as shown in the second row of Table 2. Destination Advertisement Object (DAO) is the third type of control message for which color set DAOHeader and color set DAOBaseObject is declared using the record constructor, shown in the third row of Table 2. DAO-ACK is the fourth type of control message and it is a DAO’s response. The DAOACKBaseObject color set given in the 4th row of Table 2 uses the product constructor.

#### Declarations related to network

All the color sets and their brief descriptions which are used in the network and DODAG module are given in Table 3.

**Table 3:** Declarations related to network and DODAG.

No.	Color set	Description
1	colset NetNode = record id:ID * rank:Rank;	This is a record of product of ID and Rank both are int type and give a unique identity to a node
2	colset RoutingTable = list NetNode;	All the nodes and their information collectively makes a routing table
3	colset SNetNode = product ID * Rank;	Product of ID and Rank
4	colset NetNodexRoutingTable = product NetNode * RoutingTable;	New Color Set formed with the product of NetNode and Routing table
5	colset SRouting = list SNetNode;	Somehow identical to Routing table
6	colset NodexRouting = product SNetNode * SRouting;	Node × Routing is formed to set a mechanism for the topology
7	colset NodeTopology = list NetNodexRoutingTable;	Only NodeTopology type of token are allowed for the transmission from Network to Roll
8	colset DODAGRoot = product ID * Rank * Grounded;	Root of a DODAG definition
9	colset DODAGNodes = list NetNode;	List of all nodes in that specific DODAG
10	colset DODAG = record rplInstanceID:RPLInstanceID * dodagID:DODAGID * dodagVersion:DODAGVersionID * dodagRoot:NetNode * nodes:DODAGNodes;	DODAG definition
11	colset NodexDODAGInfo = record node:NetNode * dodag:DODAG;	Node and DODAG information
12	colset NodesxDodag = list NodexDODAGInfo;	List of Node × DODAGinfo
13	var ndodag : NodesxDodag;	Variable of Node × DODAG to use on arc expression
14	var dodag : DODAG;	Variable of Node × DODAG to use on expression
15	colset ControlMsg = union DIS:DISBaseObject + DIO:DIOBaseObject + DAO:DAOBaseObject + CC:CCBaseObject + DAOACK:DAOACKBaseObject;	Union of all the five base object makes a new color set ControlMsg, which further help to format a content color set
16	colset Request = record sender:NO * target:NO;	Sender to Receiver request
17	colset Dest = union ALL + NODE: NetNode;	Union of all the available nodes makes destination
18	colset Packet = record dest : Dest * content:ControlMsg;	Which message to be sent to whom
19	colset NodexPacket = product NetNode * Packet;	This is the most critical color set which help the model to decide whether a new node will be part of network or not
20	colset RollRequest = union REQ:Request;	Union of Request color set

#### Scenario with real-time marking values

Values for the home marking are given in Table 4 to initialize the model for a real-time scenario in which packet movement is observed between nodes. The second column of the table also contains the purpose of the markings.

**Table 4:** Initial marking for objects.

Scenario	Values/Purpose
val controlMessageScenario = header = ptype = 1, code = 0, checksum = “1”, base = “1”, options = “0”	1,0,1,1,0 Control message
val distul = flag = 0, reserved = 0, options = “”	Setting the flag and reserved value to zero
val dodagScenario = rplInstanceID = 1, dodagID = 1, dodagVersion = 1, dodagRoot = id = 1, rank = 0, nodes=[]	Check by giving different values to DODAG
val daoObjectScenario = header = rplInstanceID = 1, k = true, d = true, flags = 1, reserved = 0, daoSequence = 1, dodagid = 1, options = “0”	DAO control message, different values
val dioObjectScenario = header = rplInstanceID = 1,versionNumber = 1, rank = 0, g = true, mop = 1, dodagPref = 1, dtsn = 1, flags = 1, reserved = 0, dodagid = 1, options = “0”	DIO control message, different values
val rollRequestScenario = REQ(sender = 1, target = 2)	value for unique request
val sTopology = 1‘((1,0),[])++ 1‘((2,1),[(1,0)])++v 1‘((3,1),[(1,0)])++ 1‘((4,2),[(3,1),(2,1)])++ 1‘((5,3),[(4,2)])	How directed acyclic graph proceeds with different topology values
val topologyScenario = [(id = 1,rank = 0,[]), (id = 2,rank = 1,[id = 1,rank = 0,id = 5,rank = 3]), (id = 3,rank = 1,[id = 1,rank = 0]), (id = 4,rank = 2,[id = 3,rank = 1,id = 2,rank = 1]), (id = 5,rank = 3,[id = 4,rank = 2])]	DDOAG formation with different ranks and ids
val nodepacket = (id = 1, rank = 0,dest = ALL, content = DIO dioObjectScenario)	1st variant node along with packet
val nodepacket2 = (id = 4, rank = 2,dest = NODE id = 3, rank = 1, content = DAO daoObjectScenario)	2nd variant node along with packet
val nodepacket3 = (id = 4, rank = 2,dest = ALL, content = DAO daoObjectScenario)	3rd variant node along with packet
val nodepacket4 = (id = 5, rank = 3,dest = ALL, content = DIO dioObjectScenario)	4th variant node along with packet

**Fig 4:**
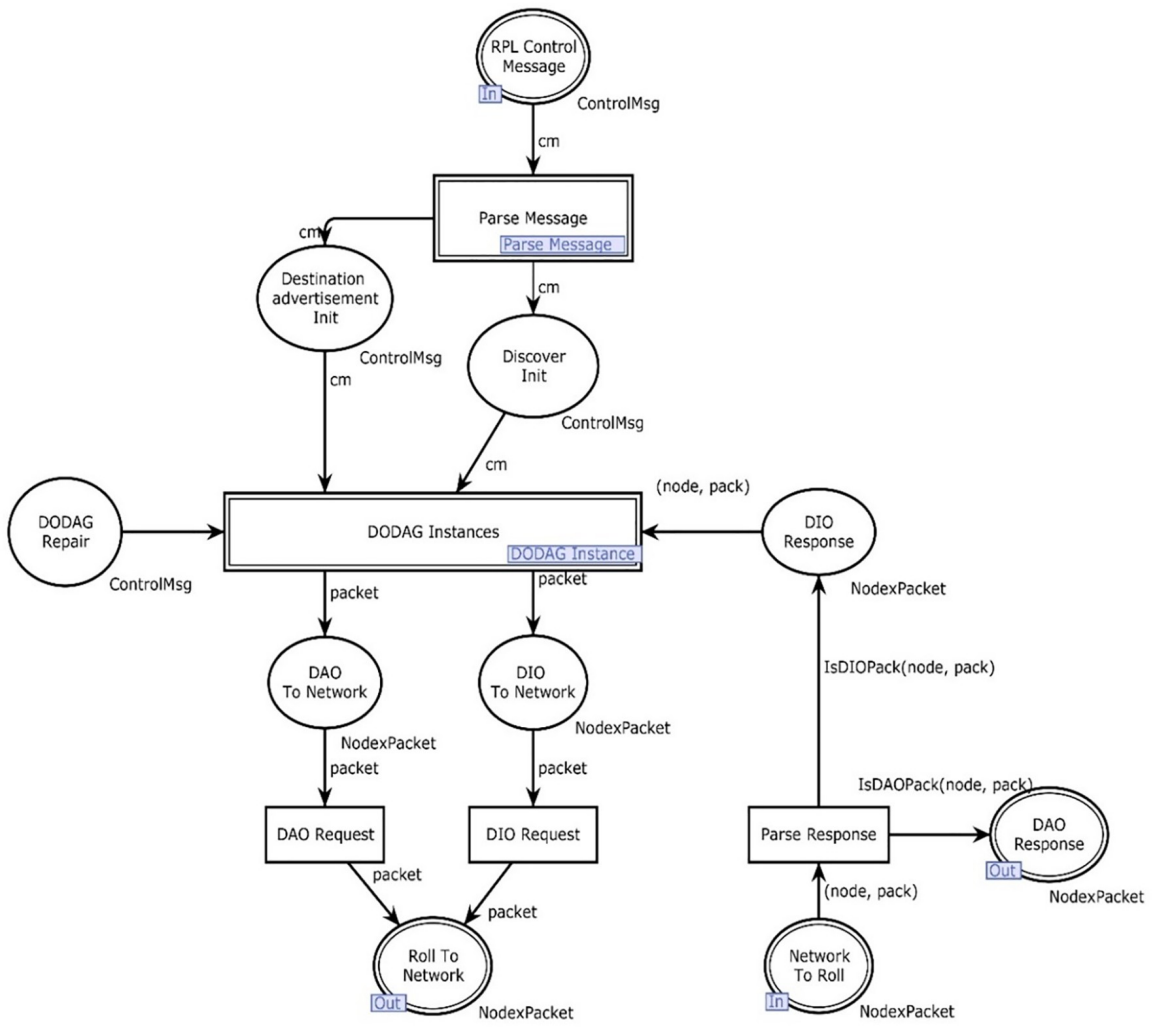
RPL instance module.

Fig 3 shows the hierarchical view of the layers in the model and Fig 4 shows an overview of the RPL CPN model structure. The model consists of two main parts, the RPL protocol model and the network. RPL instance module of the model is shown in Fig 4.

Further, two input and two output socket places of the RPL instance module of Fig 4 are described below

$$\begin{array}{c} P_{sock}^{in}\left(Roll\;Protocol\right)=\left\{RPL\;Control\;Message,\;Network\;To\;Roll\right\} \end{array}$$ (1)

$$\begin{array}{c} P_{sock}^{out}\left(Roll\;Protocol\right)=\left\{RPL\;Response,\;Roll\;To\;Network\right\} \end{array}$$ (2)

$$\begin{array}{c} P(Roll\;Protocol)=\{Destination\;advertisement,\;Disorder\;Request\} \end{array}$$ (3)

$$\begin{array}{c} T=\{Parse\;Message,\;DODAG\;Instances\} \end{array}$$ (4)

#### RPL instance module

In this section, the logical behavior of the RPL instance module is described. The protocol layer contains logic that represents the nodes in a network and what the nodes currently know about the network. It is responsible for decoding incoming control messages and encoding outgoing network messages. Fig 4 shows the representation of a DODAG instance. The network place represents the knowledge that the nodes connected to the network currently have. This includes information on the DODAG version number and ID, the RPL instance configuration, and known parent nodes and their ranks. In a typical scenario, let’s start by sending an RPL control message or a token which is given as:

*‘DIO(header = rplInstanceID = 1,versionNumber = 1,rank = 0,g = true,mop = 1, dodagPref = 1, dtsn = 1, flags = 1,reserved = 0, dodagid = 1,options=“0”)*

The control message is sent to the instance of the RPL protocol and this packet will be sent to the correct part of the model based on the message type. The protocol adds extra information to the RPL control message and sends it to the network part of the model. The packet is then transmitted over the network and back to the RPL protocol, which handles what to do with the packet.

In Fig 4, the control message enters into the parse message from RPL control messages. Since the parse message is enabled at this time, the token moves out towards the places discover Init and destination advertisement init. Firstly, the token goes to discover the request place. The DODAG instance is a hierarchal module and has a submodule of the RPL Instance. Further, the token moves inside this module. The functions used in the RPL instance module (see Fig 4) are given in Table 5.

**Table 5:** Functions used in RPL instance module.

Function	Function behavior
fun IsDIO(DIO cm) = 1‘(DIO cm) | IsDIO(cm) = empty; fun IsDIOPack(node,dest = d, content = DIO cm) = 1‘(node,dest = d, content = DIO cm) | IsDIOPack(node,dest = d, content = cm) = empty;	Checks DIO packets and send for DIO response
fun IsDAO(DAO cm) = 1‘(DAO cm) | IsDAO(cm) = empty; fun IsDAOPack(node,(dest = d, content = DAO c)) = 1‘(node,(dest = d, content = DAO c)) | IsDAOPack(node,(dest = d, content = c)) = empty;	Checks DIO packets and send for DAO response

#### RPL protocol layer

**Fig 5:**
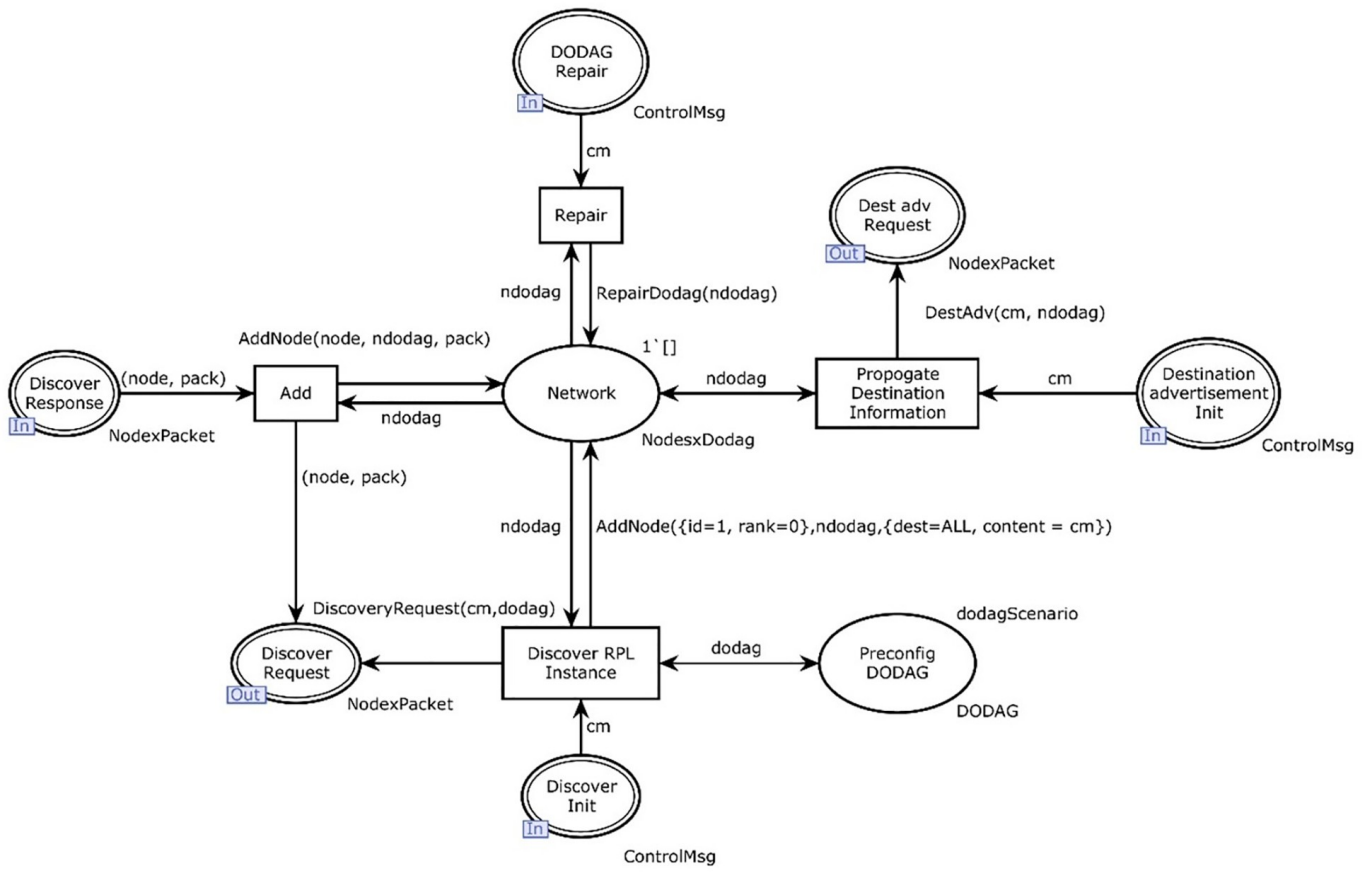
RPL protocol module.

Fig 5 contains three main parts: the logic responsible for handling the discovery of DODAGs (DIOs), the logic for destination advertisement (DAO), and a place that holds the repair of a DODAG. The discovery logic consists of initial discovery, sending of a discovery request, and a discovery response. The DAO part consists of a place creating DAO packets. DODAG repair is only a skeleton for future work and contains no logic.

The input, output places, and transitions used in Fig 5 are given below.

$$\begin{array}{c} \begin{array}{c} P_{sock}^{in}\left(RPL\;Protocol\right)=\{DODAG\;Repair,\;Discover\;Response,\\ Destination\;advertisementInit,\;DiscoverInit\} \end{array} \end{array}$$ (5)

$$\begin{array}{c} P_{sock}^{out}\left(RPL\;Protocol\right)=\left\{Dest\;adv\;Request,\;Discover\;Request\right\} \end{array}$$ (6)

$$\begin{array}{c} P=\{Network,\;Preconfig\;DODAG\} \end{array}$$ (7)

$$\begin{array}{c} T=\{Repair,\;Add,\;Propogate\;Destination\;Information,\;Discover\;RPL\;Instance\} \end{array}$$ (8)

The place preconfig DODAG contains information predefined as a scenario in our model. We have chosen to set the initial value of RPLinstanceID, DODAGID, and DODAG version number to 1. We assume that the node sending out the initial DIO request is at the root node. The root node in the DODAG is defined to have a rank of 0; in the model, the assumption is made that the DODAG root is the first to broadcast a DIO message. It is assumed that when a DIO is picked up by a node, the node joins the advertised DODAG instance. For simplicity, we say that when a specific node is receiving a DIO packet, it also sends out a DIO packet. This is not always the case, but as the current DODAG version and version number are different from what the node’s current has knowledge about (none vs 1), it will broadcast a DIO control message advertising its presence.

The network place represents the current knowledge of the nodes in the DODAG and is starting without any connected nodes. When a node joins the DODAG, it knows the RPL identifiers and tries to obtain a routing table consisting of one or more parent nodes with their associated rank. RPL protocol is the module where security risk is high. As a node is recognized by five attributes, rank, and DODAG version number are more significant attributes. Any node which is not the actual part of the specific network can change its rank or DODAG version number and may get access to the network. All those attacks which are associated with the recognition of a particular node can be detected by the proposed model and hence could be countered.

**Fig 6:**
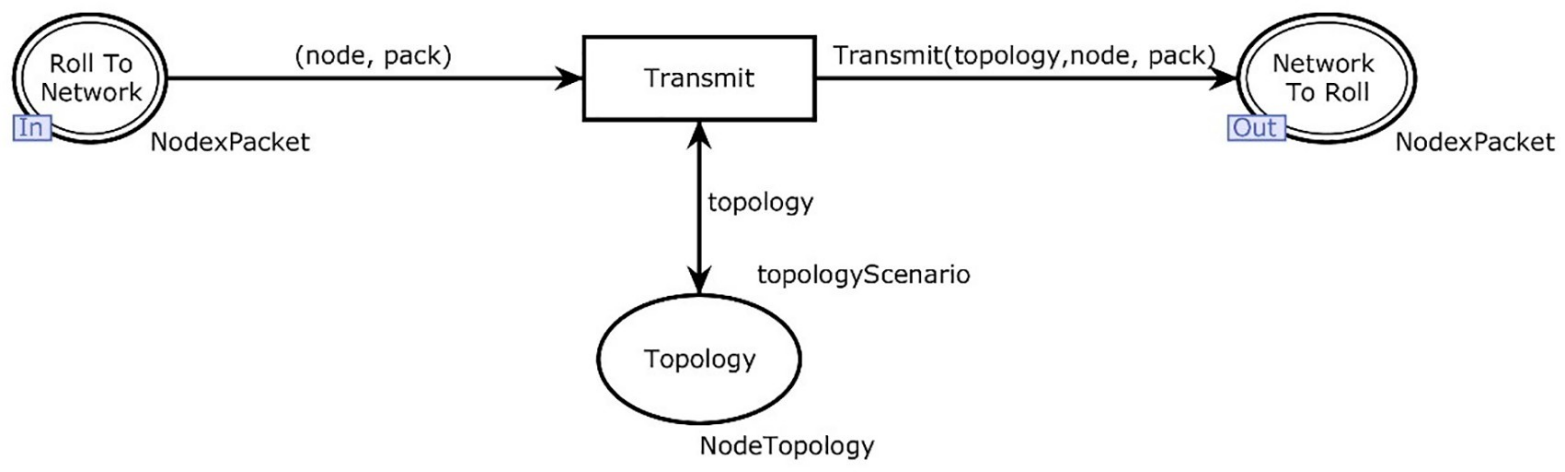
Network module.

When the token enters the roll protocol module given in Fig 5 as a DIO control message, first of all, discover RPL instance is the transition which is enabled as there is already a token in the place Preconfigure DODAG and network. As we assume there exists an already built DODAG for the first time. So, there must be a token in the network place. When this enabled transition gets fired, the token moves towards the discover request as the output place. One token also goes to the network and Preconfig DODAG each, then the control message type of token moves towards the DODAG instance module again. This is the time when the message gets ready to float over the network. At this particular marking Unicast to multicast is enabled in other words topology and hybrid place of the roll to the network have some data. At this stage, the DIO control message has been transmitted, and once again it gets ready to jump into the RPL part of our model from the network module. The marking of the network to roll place, in Fig 6, at this stage can be given by:

*<1‘(id = 2,rank = 1,dest = ALL,content = DIO(header = rplInstanceID = 1,versionNumber = 1, rank = 0,g = true,mop = 1,dodagPref = 1,dtsn = 1,flags = 1,reserved = 0,dodagid = 1,options=“0”)*) ++1‘(id = 3,rank = 1, dest = ALL,content = DIO (header = rplInstanceID = 1, versionNumber = 1,rank = 0,g = true,mop = 1, dodagPref = 1,dtsn = 1, flags = 1, reserved = 0,dodagid = 1,options=“0”))>.

Parse response transition gets enabled mode and notes that the place network to roll has exactly two modules in it. On firing, one token moves to the DIO response, and one token gets access to the RPL instance module. At this stage DoDAG instance, the place is not enabled because of the emptiness of its other input place. In RPL instance module critical transition add gets enabled as the network and discover response has exactly one token. The token moves to the network and discovers a request for firing. DIO to network once again gets one token and the DIO request gains the status of the enabled transition. At the current phase of the running module, the packet moves toward a discovered response. The place discovers response helps the packet add information of node to it and a new node is added and on firing one token moves to discover request. At this stage, the discover request has two tokens in it of node* pack type. Node*packet moves through the DIO request. This particular type of toke is now ready again to jump over the network. In Fig 6, the transmit transition of the network module is enabled now to transmit the packet with some additional information multipoint to multipoint at this time. This is how a directed acyclic graph is formulated. Table 6 presents the functions used in the RPL protocol module.

**Table 6:** Functions used in RPL protocol module.

Function	Function behavior
fun DestAdv(cm, dodag) = 1‘(id = 1, rank = 0, dest = ALL, content = cm)	Advertisement of destination by a node
fun AddNodeToDag(node, rplInstanceID = id, dodagID = did, dodagVersion = dv, dodagRoot = dr, nodes = ns) = 1‘(rplInstanceID = id, dodagID = did, dodagVersion = dv, dodagRoot = dr, nodes = (node::ns))	Add node to sub DODAG
fun AddNode(n, ndodag, dest = d,content = DIO(header = rplInstanceID = rplid,versionNumber = vn, rank = rnk,g = ground, mop = mops, dodagPref = dodpref, dtsn = dtsn, flags = flags, reserved = reserved, dodagid = dodid, options = opts)) = if (List.exists (fn node = no,dodag = dod => (no = n)) ndodag) then sort_ms NodexDODAGInfo.lt ndodag else sort_ms NodexDODAGInfo.lt (node = n,dodag = rplInstanceID = rplid, dodagID = dodid, dodagVersion = vn, dodagRoot = id = 1, rank = 0, nodes = []::ndodag);	Add new as a child node if previous checks meets
fun DiscoveryRequest(cm, rplInstanceID = id, dodagID = did, dodagVersion = dv, dodagRoot = dr, nodes = ns) = 1‘(dr,dest = ALL,content = cm);	Enable Neighbored announcement
fun Discover(topology, node, cmsg) = if topology = [] then empty else let val rtable = GetRtable(List.hd topology) val nnode = GetNode(List.hd topology) in if (List.exists (fn d => (d = node)) rtable) then 1‘(nnode, cmsg)++Discover(List.tl topology, node, cmsg) else Discover(List.tl topology, node, cmsg) end	Upward route discovery allows a node to join a DODAG by discovering neighbors that are members of the DODA
fun RepairDodag(dodag) = dodag;	Repairing DODAG (future extension)
fun DAOAck(dao, dodag) = (1, true, 1, 1, 1, 1, “ok”) Acknowledgement function	Acknowledgement function

#### Network layer

The network will receive an arbitrary RPL control message and based on the type and destination, it figures out what to do. When a DAO with a ‘dest = Node *n*’ is received, it will dispatch those packets toward the destination nodes if it is reachable or disregard them if not. The DAO control message is defined as follows by the RPL RFC [8].

**Destination advertisement object**: The destination advertisement object (DAO) is used to disseminate information upward in the DODAG. The DIO message works slightly differently. A DIO message is broadcasted, and the nodes that have the sender as a parent will get the message. The DIO message cannot be unicasted as the node sending the DIO will not have any information about nodes further down in the DODAG. The DIO is broadcasted and transmitted downward in the DODAG, as opposed to DAO messages which are unicast from one node and upwards to one or more of its known parents.

Fig 6 shows the representation of a physical network model using CPN tools. The network is given a node that represents the sender and a packet containing a destination and a control message object. The place topology in Fig 6 represents the actual topology of the current network and is predefined.

The input, output places, and single transition of Fig 6 are defined below:

$$\begin{array}{c} P_{sock}^{in}\left(Network\right)=\left\{Roll\;To\;Network\right\} \end{array}$$ (9)

$$\begin{array}{c} P_{sock}^{out}\left(Network\right)=\left\{Network\;To\;Roll\right\} \end{array}$$ (10)

where *P* is the topology and *T* is the transmit. The socket of the network in the main module has only place denoted by roll To network and so is outgoing. In a higher abstraction, the module contains one place and one transition known as topology and transmit. Further, Table 7 presents the function used in the network module.

**Table 7:** Function used in the network module.

fun Transmit(topology, node, dest = ALL, content = DIO cm) =
if topology = [] then empty
else
let
val rtable = GetRtable(List.hd topology)
val nnode = GetNode(List.hd topology)
val cmsg = dest = dest, content = DIO cm
in
if (List.exists (fn d => (d = node)) rtable) then
1’(nnode, cmsg)++Transmit(List.tl topology, node, cmsg)
else
Transmit(List.tl topology, node, cmsg)
end

Literature suggests the mitigation technique at the topology level. Based on that mitigation, the declarations given in Table 7 are part of the function that describes how packets are transmitted. It matches control messages that are of type DIO and that are being broadcasted. When this is the case, it looks in the topology for nodes that have the sender (node) as a parent and sends the DIO message to those nodes.

The transmission of packets is based on matching the content type of the control message and destination. In the case of a DIO message, it will go downwards in the DODAG, and not upwards. This means that the message cannot simply be modeled as a broadcast message, because the routing table only contains the parents of a node, not the children. This function goes through the topology and sends the message to whoever has the node sending out the DIO as a parent.

### Overview of RPL attacks

It is anticipated that the quantity of associated machines is evaluated to develop exclusively as much as 55 billion in a few years. The developing enthusiasm for IoT is adding to the enormous scale organization of LLN. This network usually bolsters communication between sensor devices. So, the said protocol has opened the door for different kinds of adversaries. In recent research, numerous works examined the performance and security aspects of the different protocols being used in IoT; for example, studies performed some sort of simulations for assessing the performance [53–55], and [56]. In [53], the researchers just proposed an upgraded adaptation of protocol for LLN for IoT. [55] suggested another validation protocol for mobile IoT to build a safe system. This protocol was well tried and tested and has some simulations.

### Security of RPL protocol

There are three modes of RPL protocol described by the IETF ROLL working group [RFC 6550].

**Unsecured mode**. This mode comprises only the security level that is provided by the link layer which means all messages are not sent without any protection.

**Pre-installed mode**. In this mode of RPL, secure messages are propagated. To be a part of a new RPL instance a node should have already installed a key on it. Nodes utilize it to give message secrecy. A node can be a part of RPL and it can play the role of a host or a router by using this pre-installed key.

**Authenticated mode**. To be a part of a new RPL Instance, a node should have already installed a key on it. In this mode, nodes utilize it to give message secrecy also. A node can be a part of RPL and it can play the role of a host at a time. To be a part of the network as a router node must have to get another key for authorization.

### Modeling of pre-installed mode

**Fig 7:**
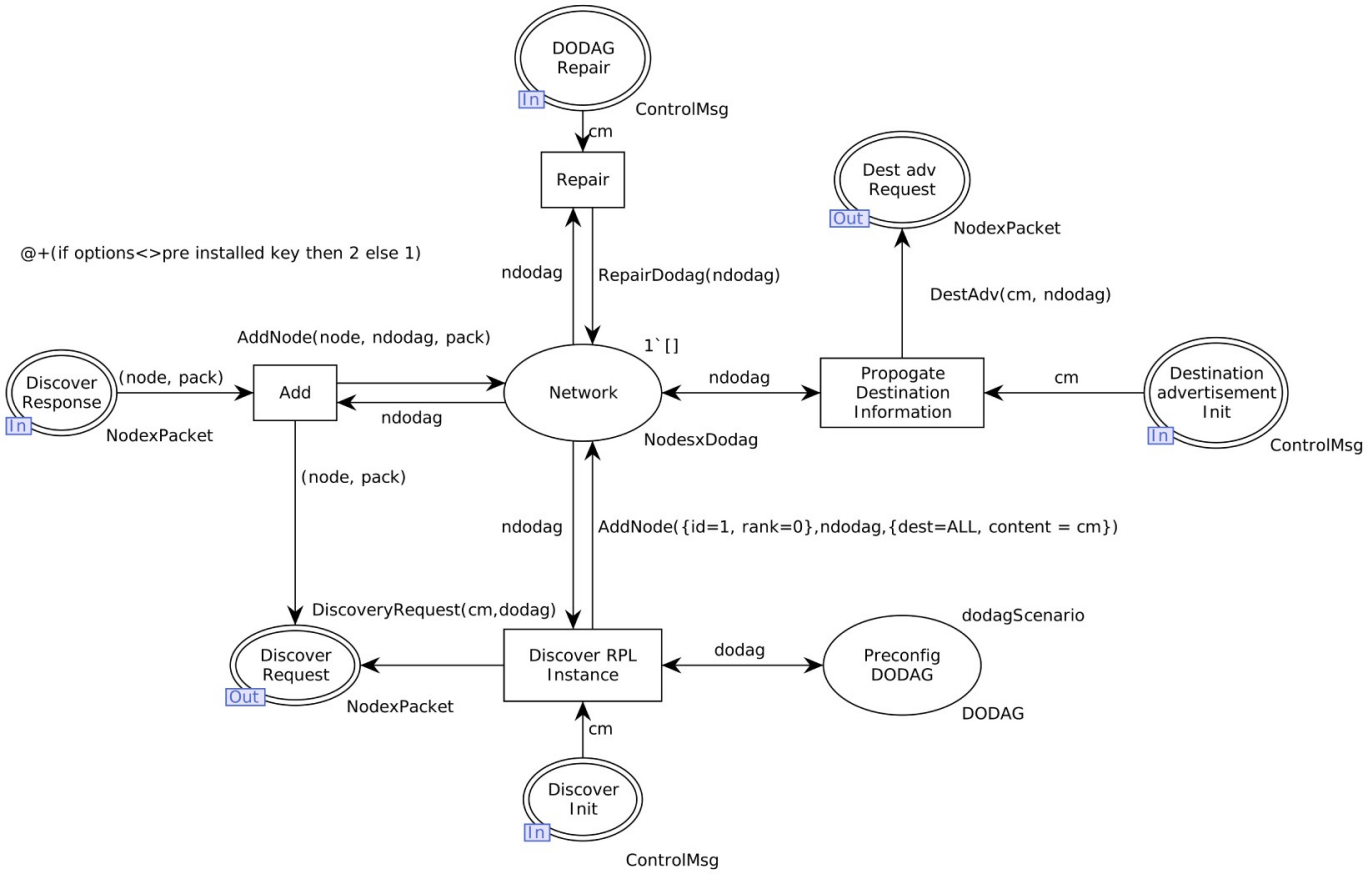
RPL instance of secure mode model.

All the color sets which are necessary to initialize the modal as a secure model are given in Table 8. The use of these color sets can be seen in the model given in Fig 7 which now has a new guard on the ‘Add’ transition. Before adding the new node, the guard is executed only if the value of options is equal to preinstalled keys. If the guard is not satisfied, the CPN model will not allow moving further, and the simulation stops. The value of the option comes from the definition of the controlled message and is checked with the preinstalled key at this stage. When the control message satisfies the guard, it will move to the network part for transmission after the addition of the node.

**Table 8:** Color sets to initialize secure mode.

Color sets in the model for security	ICMPv6 RPL control message characteristics
colset SecurityHeader = product T* Reserved * Algorithm * KIM * Resvd * LVL * Flags;	the security header carrying a control message.
colset Security = product SecurityHeader * Counter * KeyIdentifier;	Defines how message counter is used with security header and key identifier
colset CMHeader = record ptype:Type * code:Code* checksum:Checksum;	The control message header is defined by code or message body and checksum
colset ControlMessage = record header:CMHeader * base:Base * options:Options;	Saves control message with different options
colset SecureControlMessage = product CMHeader * Security * Options;	Defines a complete secure message with options

#### Version number attack

In the RPL protocol, the DODAG version number is associated with DIO messages. The root node of a specific DODAG can only change the DODAG version number and it is then attached to all the other nodes of this DODAG. If a malicious node forcefully changed the DODAG version number of a DODAG and then all the other nodes start sending the messages to this node. So, the adversary can suck the considerable resources of a network and may cause a deterministic role in the efficiency of a network [57].

#### Behavior of pre-installed mode model

The unsecure mode of RPL depends upon the security provided by the Link Layer of the IP stack. Hence the DODAG version number attack is possible in the unsecure mode. We demonstrated this by introducing a change in the DODAG version number of DIO control message so that the simulation would go through the global repair operation. As there is no security mechanism in unsecure mode to curb this attack, no exceptions were observed during the simulation.

Next, we implemented the secure/pre-installed mode by setting the packet security section to use the following pre-installed key value for authentication check:

<*pre_installed_key* = “0 × 36,0 × 54,0 × 69,0 × 53,0 × 43,0 × 48,0 × 20,0 × 6*D*,0 × 69,0 × 6*E*,0 × 69,0 × 6*D*,0 × 61,0 × 6*C*,0 × 31,0 × 35*a*”;>

Then we introduced the variations in the key and DODAG version number. As expected, the pre-installed mode simulation had a premature end until it reached the guard of add a transition. Thus the pre-installed mode proved its robustness against the unauthorized key variation and DODAG version number change as such from a malicious node. The time parameter has also significant importance here. This implies the basic concept of computer science which is the trade-off between time and complexity/size. By adding the guard condition there is a deliberate little delay in the packet delivery. An interesting modification in this model, Node×Packet is a timed color set now. The transition add has a delay equal to <@+(if options<>pre_installed_key then 2 else 1)>. In this mode packet reaches with the time stamp and significant changes in time could be noted easily to check the consistency of messages.

After the analysis of the model, the incorporation of the capacity constraint loop in the model can be proposed. However, since this specification is not mentioned in the RPL standards RFC [6550] the impact on the resources is not known. During the model development, we have to keep in mind that this protocol has constraints on resources like energy.

### State space-based analysis

State-space analysis of secure mode model with guard and matching pre-installed key is given in Table 9. Simulation results of replications through the monitors of the data collector and marking size at the transition transmit and NetworkToRoll place is performed. Five replications of the secure mode given in Fig 7 are performed which is achieved through the auxiliary text <Replications.nreplications 5> and evaluating it as ML expression. When there is no conflict in preinstalled key and options models, the simulation is performed for millions of steps until the stop criteria are met.

**Table 9:** State-space analysis of secure mode model, as given in Fig 7.

	State Space	Scc Graph
Nodes	208	2
Arcs	405	1
Secs	0	0
Status	Full	-

State-space analysis of the secure model with modified DODAG version number and the non-matching pre-installed key is shown in Table 10. It can be seen clearly when an attacker attempts to join the network by pretending a real DODAG version number, the model denies it and stops the simulation.

**Table 10:** State-space analysis of secure mode model in Fig 7 under attack.

	State Space	Scc Graph
Nodes	1	1
Arcs	0	0
Secs	0	0
Status	Full	-

### Practical applications of proposed approach

The good impact applications of the proposed approach are related to multiple areas of computer science such as network security vulnerabilities analysis and prediction, data security evaluation for IoT-related edge computing, cloud workload scheduling, etc. We have proposed a CPN-based simulator that can be as useful as any other programming/ simulation environment for all formally representable phenomena.

### Limitations of formal modeling

The proposed work is based on the formal technique and one of the main limitations of using formal techniques to model large-scale complex systems is to compromise on the readability and understandability of the formal model of the system. Moreover, the formal model of a complex system becomes so large and complex that may lower its understandability. Furthermore, formal analysis and validation of large-scale formal models of the systems are challenging and face the famous state-space explosion problem [15]. However, to alleviate this limitation, we adopted the hierarchical colored Petri nets that help in developing modules of large-scale systems to give a compact representation of the system model. Further, hierarchical-colored Petri nets use CPN ML language (which is based on functional programming language i.e., Standard ML) to model complex systems. Therefore, the main limitation of modeling large-scale systems can be tackled through CPN-based formalism.

### Discussions

The proposed system has the scope of bootstrapping, communication, and security evaluation of the secure mode of RPL. As mentioned in Sections 4 and 5, the modeling of RPL in the proposed work is as close to real-life working as possible in terms of network concepts. Hence (to the best of our knowledge) the computational complexity of bootstrapping and communication is in parallel to the RPL pre-installed mode and for any other simulation environment. For communication cost, the implementation of variety in network channel conditions such as channel capacity and noise is regarded as part of future implementation regarding the benchmarking and stress testing of RPL. For security, the secure mode is implemented in the proposed work and it has been tested for vulnerability in Section 5.6. The security checks are implemented as per the RPL standards RFC [6550] and as shown in Sections 5.6 and 5.7, the CPN model behaves accordingly with minimum or no deviation from the original RFC.

## Conclusions

A colored Petri net-based formal model of routing over LLN has been presented in this research. Modeling the RPL protocol in CPN tools has given a lot of insight into how the RPL protocol works, but the protocol specification is quite large and detailed so a complete overview is hard to get. Therefore, the research proceeded towards developing the module where control message can exceed their limits. Moreover, the focus was shifted towards the evaluation of the existing security standards for RPL through formal validation and verification to check if those standards can achieve the desired security level. The state-space analysis shows that the model ends up in the desired state when there are no loops in the graph representing the network. The loop discovery and inconsistency repair are not implemented in the model and an incorrect topology will therefore cause undesired results. The state space reveals a smaller amount of information about the protocol as a whole from the subset we have chosen to model. The simulation and state-space analysis do however indicate that the distributed algorithm operation works as expected. Simulations show the smooth flow of packets when there is no mechanism to detect and stop the adversaries in the system. When a secured key mechanism is applied to the system, the system stops the spontaneous flow of packets. In the future, we plan to incorporate the security attacks mentioned in [58–60] and provide a formal analysis of remedies to the attacks as discussed in the literature. Furthermore, we plan to incorporate mobility management in RPL and the various extensions of RPL, as mentioned in [61].
